# Technical note: Experimental determination of the effective point of measurement of the PTW‐31010 ionization chamber in proton and carbon ion beams

**DOI:** 10.1002/mp.15377

**Published:** 2021-12-15

**Authors:** Sandra Barna, Andreas Franz Resch, Monika Puchalska, Dietmar Georg, Hugo Palmans

**Affiliations:** ^1^ Department of Radiation Oncology Medical University of Vienna Vienna Austria; ^2^ Atominstitut Technical University of Vienna Vienna Austria; ^3^ MedAustron Ion Therapy Center Wiener Neustadt Austria; ^4^ National Physical Laboratory Teddington UK

**Keywords:** dosimetry, effective point of measurement, particle beams

## Abstract

**Purpose:**

The accurate knowledge of the effective point of measurement (*P*_eff_) is particularly important for measurements in proximity to high dose gradients such as in the distal fall‐off of particle beams. For plane‐parallel ionization chambers (ICs), *P*_eff_ is well known and located at the center of the inner surface of the entrance window. For cylindrical ICs, *P*_eff_ is shifted from the chamber's center toward the beam source. According to IAEA TRS‐398, this shift can be calculated as 0.75·*r*_cyl_ for light ions with *r*_cyl_ being the radius of the cavity. For proton beams and in absence of a dose gradient, no shift is recommended. We have experimentally determined *P*_eff_ for the 0.125 cc Semiflex IC in both proton and carbon ion beams.

**Methods:**

The first method consisted of simultaneous irradiation of a plane‐parallel IC and the Semiflex in a 4‐cm wide spread‐out Bragg peak. In the second method, a single‐energy beam was used, and both ICs were positioned successively at the same measurement depths. For both approaches, the shift of the distal edge of the depth ionization distributions recorded by the two chambers at different reference points was used to calculate *P*_eff_ of the Semiflex. Both methods were applied in carbon ion beams, and only the latter was applied in proton beams.

**Results:**

Both methods yielded a similar *P*_eff_ for carbon ions, 0.88·*r*_cyl_, and 0.84·*r*_cyl_, which results in a difference of only 0.1 mm. The difference to the recommended value of 0.75·*r*_cyl_ is 0.4 and 0.3 mm, respectively, which is larger than the positioning uncertainty. In the proton beam, a *P*_eff_ of 0.92·*r*_cyl_ was obtained.

**Conclusions:**

The *P*_eff_ for the 0.125 cc Semiflex IC is shifted further from the cavity center as recommended by IAEA TRS‐398 for light ions, with the shift for proton beams being even larger than for carbon ion beams.

## 1 INTRODUCTION

The measurement of depth dose distributions is one of the key tasks during commissioning of light‐ion beams for radiotherapy. Plane‐parallel ionization chambers (PPICs) are usually preferred for this purpose, but small cylindrical ICs (CICs) can also be used. The first category of ICs has the advantage of a high spatial resolution in depth and a well‐defined effective point of measurement (*P*_eff_), which is located at the center of the inner surface of the entrance window. On the other hand, they have two main disadvantages: (1) The polarity effect can be substantial in regions where charged particles stop and can vary rapidly with local changes of the charged particle spectrum, and (2) the alignment of the cavity's symmetry axis with the beam axis is critical and not trivial to achieve; any misalignment will lead to a blurring of the measurement depth and, for example, result in a widening of the measured Bragg peak and distal edge. CICs have the advantage of generally exhibiting lower polarity effects, and because of their rotational symmetry, alignment is less critical. The disadvantage of CICs is that an averaging effect takes place with particles that enter the cavity at different points having different expectation values of energy loss history. This averaging effect results in a *P*_eff_, that is, with respect to the center of the chamber, shifted toward the beam source.

A document has been released by the International Atomic Energy Agency (IAEA) as part of their technical report series (TRS) on the code of practice regarding dosimetry. This document, IAEA TRS‐398^1^, recommends a *P*_eff_ for CICs in light ion beams located 0.75·*r*_cyl_ closer to the beam source as the center of the IC, with *r*_cyl_ being the radius of the cavity. For proton beams, the center of the chamber is used as the reference point which is justified since dosimetry is recommended to be performed in the middle of the spread‐out Bragg peak (SOBP). No recommendation for the use of CICs in proton beams in the presence of depth dose gradients is made.

Looe et al.^2^ investigated the shift of the effective point of measurement of several CICs and PPICs in electron and photon beams, among others, the 0.3 cc Semiflex IC. Only few experimental investigations have been performed in light ion beams on this subject and none with non‐Farmer‐type IC. *P*_eff_ was determined for Farmer‐type ICs in protons^3^ and in carbon ions.^4^ Overall, these data are all consistent with the value of 0.75·*r*_cyl_, but there is considerable spread, and a standard deviation of 0.3 mm was deducted by Palmans.^5^

In general, CICs offer advantages in experimental set‐ups with limited space or for specific dosimetric purposes. For example, because of the 0.125 cc Semiflex's convenient size and waterproofness, it can be easily inserted in a biological flask for commissioning of cell irradiation set‐ups, and thus *P*_eff_ effects might have impact on reference dosimetry in the context of radiobiological experiments. In accordance with the report of a National Cancer Institute special panel,^6^ high quality physics parameters should be provided for pre‐clinical research, which implies as low as possible dosimetric uncertainties. An interdisciplinary collaboration for radiobiological cell irradiation with proton and carbon ion beams, which are often based on in‐house‐developed solutions, motivated this study.

As for the 0.125 cc Semiflex IC, no experimental data are available for light ion beams, the aim of our study was to experimentally determine *P*_eff_ for this widely used IC in both proton and carbon ion beams.

## 2 MATERIALS AND METHODS

The 0.125 cc Semiflex chamber was investigated, which has a thimble cavity that forms an almost spherical sensitive volume of 0.125 cm^3^. The radius of the cylindrical part of the cavity is 2.75 mm, and the length is 6.5 mm. No build‐up cap was used. As a reference, an Advanced Markus chamber, a PPIC with a nominal sensitive volume of 0.2 cm^3^ and a radius of 2.5 mm, was used.

Both ICs were cross‐calibrated against a Farmer reference class IC in the entrance region (low dose gradient) of a 179.2 MeV proton beam and a 346.6 MeV/n carbon ion beam. Therefore we assumed a displacement correction factor *k*_Q_ = 1, given the small variation of *k*_Q_ with beam quality. For the Farmer chamber, which has a calibration coefficient traceable to the Austrian National Measurement Institute (Bundesamt für Eich‐ und Vermessungswesen [BEV]), we used the *k*_Q_ given in IAEA TRS‐398 (*k*_Q_ = 1.029 for protons and *k*_Q_ = 1.032 for carbon ions).

Two different experimental set‐ups were utilized (see Table 1). For the gradient method, a carbon ion treatment plan was calculated using the treatment planning system (TPS) RayStation v7.99 PB v3.0. A 4 cm SOBP was created to deliver a homogeneous physical dose of 0.5 Gy using several energies with a maximum beam energy of 231.2 MeV/u. In the peak‐to‐peak method, a 172.9 MeV per nucleon single‐energy beam was used for carbon ions and a 90.4 MeV beam for protons.

**TABLE 1:** Equipment used during both experimental set‐ups, purchased from PTW Freiburg, Germany

Equipment	Gradient method	Peak‐to‐peak method
CIC	0.125 cc Semiflex chamber (Type 31010, SN 006012)	
PPIC	Advanced Markus electron chamber (Type 34045, SN 001540)	
Water phantom	Water phantom 41023 for horizontal beams	MP3‐P water phantom system
Electrometer	UNIDOS webline	

Abbreviations: CICs, cylindrical ionization chambers; PPICs, plane‐parallel ionization chambers.

According to IAEA TRS‐398, adequate positioning of PPICs takes the water‐equivalent thickness (WET) of the entrance wall of the chamber into account. However, this is often approximated by using the physical density leading to minor errors of 0.1–0.2 mm for most commercial PPICs. For the Advanced Markus IC, the total window area density including the protection cap is given as 1.06 g/cm^2^. However, it needs to be considered that this value is recommended for photons. We used the relative WET (rWET) values published by Lourenço et al.^7^ for protons and assumed the same rWET values for carbon ions as well. The protection cap (PMMA, 1.19 g/cm^2^) and the entrance foil (polyethylene [PE], 2.76 g/cm^2^) have a respective thickness of 0.87 and 0.03 mm. With rWET(PMMA) = 1.1635 and rWET(PE) = 0.9958, the WET of the Advanced Markus chamber is equal to 1.04 mm for light ions.

### 2.1 Experimental set‐up: Gradient versus peak‐to‐peak method

In the gradient method, measurements were performed in a stationary water phantom with customized holders for cell experiments. The mechanical scale of this water phantom allows positioning with a resolution down to the 0.1‐mm level, and its absolute accuracy was established as 0.2 mm. The phantom was filled with distilled water 1 h prior to measurements allowing for the window deflection to settle. The window thickness is equal to 0.305 mm, resulting in a WET of 0.355 mm using the previously introduced rWET (PMMA).

The ICs were fixed inside flasks for biological experiments (15‐ml Slide Flask, Thermo Fisher Scientific, Langenselbold, Germany), and the flasks were in turn fixed inside the customized set‐up about 3 cm apart. The thickness and material of the separated slide are given as 1.3 mm PE by the manufacturer. We measured a thickness of 1.33 mm and 1.34 mm in the chamber slides modified to fit the PPIC and CIC, respectively.

The second set of measurements for the peak‐to‐peak method was performed in a motorized water phantom. The WET of the window is equal to 5.84 mm and was measured during commissioning procedures. The ICs were fixed with their assumed *P*_eff_ at the depth of interest. For the CIC, *P*_eff_ = 0.75·*r*_cyl_ = 2.06 mm according to IAEA TRS‐398, while for the PPIC, the previously established WET of 1.04 mm was assumed. The positioning of the CIC and PPIC relative to each other is schematically visualized for both methods in Figure 1.

**FIGURE 1:**
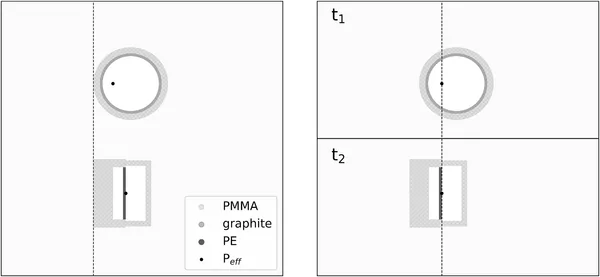
Schematics of the relative positions of the ionization chambers (ICs). The different elemental components are not to scale (left). The cylindrical ionization chambers (CICs) and plane‐parallel ionization chambers (PPICs) were placed inside chamber slides and aligned by the outer wall of the sensitive volume, not by their *P*_eff_, allowing for simultaneous irradiation of both ICs (right). The CIC and PPIC were aligned with respect to their (assumed) *P*_eff_ and irradiated sequentially. They were irradiated consecutively at different time points t_1_ and t_2_ minutes apart

### 2.2 Measurements: Gradient versus peak‐to‐peak method

For the gradient method, 14 points on an SOBP were measured thrice with both ICs in parallel in the stationary water phantom; its entrance window was aligned with the room's isocenter prior to any measurements. Five of those points were in a relevant range of the dose fall‐off with an equidistant spacing of 0.1 mm. Two 4th‐order polynomials were fitted to the 0.125 cc Semiflex and Advanced Markus IC data points. The inflection point on the Advanced Markus’ polynomial fit as well as R80 and R50 (ranges at 80% and 50% of the maximum dose) was used as reference for the shift between the CIC of unknown *P*_eff_ and the PPIC of known *P*_eff_. This method is based exclusively on the fall‐off region, that is, a high gradient region, hence the name “gradient method.”

For the peak‐to‐peak method, 28 and 42 points were measured along depth dose profiles in a 90.4 MeV proton and 172.9 MeV/u carbon ion beam, respectively. Both irradiation set‐ups used a 3‐cm PMMA range shifter, as well as a brass collimator with 15‐mm aperture diameter which was placed at the room's isocenter. The motorized water phantom was placed after a 5‐cm air gap. Each point was measured once, based on the previously established stable response of both ICs. The difference between the peak positions as well as R80 and R50 was used to determine the difference in (assumed and known) *P*_eff_ between the 0.125 cc Semiflex and Advanced Markus IC.

Both methods as well as the measured dose along the central axis in beam direction can be seen in Figure 2.

**FIGURE 2:**
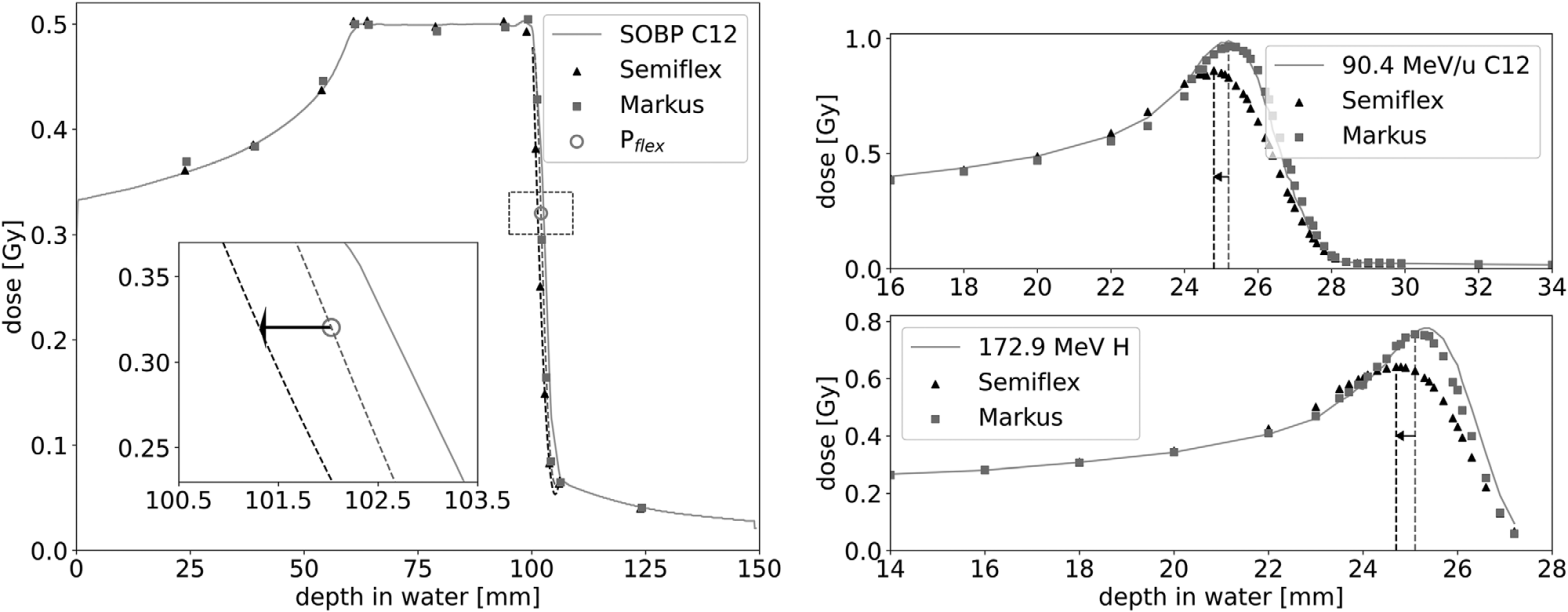
Depth dose curves (left). The gradient method, visualizing the shift between the polynomial fit of the 0.125 cc Semiflex and Advanced Markus IC fall‐off data points at the inflection point *P*_flex_ (right). The depth dose curves were derived with Monte Carlo simulations using GATE/Geant4 and a previously validated beam model of our facility's nozzle design.^8^ The peak‐to‐peak method is visualized by the shift between the maximum dose measured with the 0.125 cc Semiflex and Advanced Markus IC

### 2.3 Validation of measurements with gamma analysis

To demonstrate the non‐negligible impact of the derived *P*_eff_ compared to the IAEA TRS‐398 recommendation, a gamma‐index‐analysis^9^ in 1D was performed to compare the measured depth dose distribution, and the TPS calculated depth dose curve. The derived *P*_eff_ for the 0.125 cc Semiflex was applied to the gradient method's measurements with carbon ions as well as protons. No SOBP proton measurements were used in this study due to a lack of data points in the fall‐off region, which makes it an independent dataset to test our result on. The voxel size in the TPS was 1 mm, which motivated the choice of rather strict acceptance criteria Δd/ΔD equal to 1 mm/1% for carbon ions and 1 mm/2% for protons. The slightly more lenient dose criteria for protons are due to fluctuations in the SOBP.

### 2.4 Uncertainties in the measurements

The type A uncertainty^10^ derived from the gradient method measurements was assumed to be representative for the peak‐to‐peak method, as the exact same ICs and beam line were used. The mean over the fractional uncertainty of the five points in the fall‐off was 0.43% for the Semiflex measurements and 0.34% for the Advanced Markus measurements. This translated to an uncertainty in the depth of 0.01 mm, using the reciprocal of the depth dose gradient at R80 as a sensitivity coefficient.

Several factors contributed to the type B uncertainty; an overview is given in Table 2. The scale of the stationary water phantom as well as the motorized water phantom allows adjustments down to 0.1 mm. Assuming a rectangular distribution with a width of 0.1 mm, the associated uncertainty is 0.06 mm. The side‐by‐side measurement arrangement of the gradient method is susceptible to tilts, which were estimated to be of the order of 1 mm in horizontal and vertical direction. Both would cause a misalignment of the IC of 1 μm in depth, which we considered negligible. The previously described general positioning accuracy of the detector relative to the scale was determined to be 0.2 mm for the gradient method. When commercial IC holders were used, relative positioning in relation to the window of the water phantom was performed which is associated with a standard uncertainty of 0.15 mm. The used rWET values are associated with a 1% combined uncertainty by the publishing authors,^7^ this contribution was considered negligible.

**TABLE 2:** Contributions to the experimental standard uncertainty of the relative position of both ionization chambers

Source of uncertainty	Gradient method (mm)	Peak‐to‐peak method (mm)
Type A		
Reproducibility	0.01	0.01
Type B		
Resolution of scale	0.06	0.06
Positioning Semiflex	0.20	0.15
Positioning Advanced Markus	0.20	0.15
Total	0.29	0.22

The general positioning uncertainty of the customized holder for biological experiments was estimated with the following investigation. The customized holder introduces a non‐standard offset from the water phantom scale. This offset was determined using a parallel gauge block set (VOGEL Germany). The offset from the scale has been calculated as the mean over measurements done by four researchers on 2 different days to estimate repeatability and reproducibility. By applying a triangular distribution and taking the extreme values as the full width at half maximum, the offset was determined within 1*σ* = 0.2 mm. The offset introduced by the customized holder was identical for both ICs and therefore does not contribute to the uncertainty of the measured *P*_eff_ assuming total correlation.

## 3 RESULTS AND DISCUSSION

In Figure 2, a difference between the slopes of the pristine Bragg peak measured with the Advanced Markus versus 0.125 cc Semiflex IC can be seen. This distortion of the Bragg peak by CICs in general was previously described by Palmans^11^ and is due to the energy fluence averaging effect. We have compared the slope of the two polynomials fitted to the dose fall‐off measurements performed in the SOBP with both ICs and found no substantial difference.

### 3.1 Measurements: Gradient versus peak‐to‐peak method

The results of the gradient and peak‐to‐peak method for different reference points (inflection point, peak position, R80, and R50) are summarized in Table 3. Regarding carbon ions, good agreement was found between the two methods used to determine *P*_eff_ of the 0.125 cc Semiflex IC. The difference between the *P*_eff_ established by the gradient and peak‐to‐peak method equals 0.1 mm. The consistent result between a measurement done in a biological flask and in open water shows that a *P*_eff_ determined in water can be used for biological experiments as well.

**TABLE 3:** Factors to be multiplied with *r*_cyl_ derived from measurements in a spread‐out Bragg peak and pristine Bragg peak for light ions

	Gradient method – carbon ions	Peak‐to‐peak method – carbon ions	Peak‐to‐peak method – protons
R50	(0.84 ± 0.11)·*r*_cyl_	(0.79 ± 0.08)·*r*_cyl_	(0.86 ± 0.08)·*r*_cyl_
R80	(0.88 ± 0.11)·*r*_cyl_	(0.84 ± 0.08)·*r*_cyl_	(0.92 ± 0.08)·*r*_cyl_
Inflection point	(0.84 ± 0.11)·*r*_cyl_	–	–
Peak position	–	(0.89 ± 0.08)·*r*_cyl_	(0.96 ± 0.08)·*r*_cyl_

As discussed by Palmans,^11^ the effective point of measurement depends on the depth and is not a constant factor. Palmans concluded that the shift of the distal edge gives a good estimate for the whole depth dose curve, except around the Bragg peak if its distortion is substantial.

### 3.2 Comparison with literature

The analytical method of Palmans^12^ results in 0.74·*r*_cyl_, which is almost identical to the IAEA TRS‐398 recommendation. This is a difference of 0.3 mm for carbon ions and 0.5 mm for protons, which are in the order of the measurement uncertainty for carbon ions and larger than the measurement uncertainty for protons. This may be explained by the simplistic nature of the analytical model, as it assumes a straight particle track parallel to the beam axis and does not distinguish between different particle types. Farmer type chambers have a larger volume and a thin electrode, causing a larger section of the volume to be at a similar effective depth. For smaller ICs, the electrode in relation to the volume is bigger, and therefore a large fraction of the volume is at the periphery of the chamber, where scatter contributions get more important to consider. This indicates a limitation to the analytical model for smaller CICs. Another reason could be the geometrical shape, as the 0.125 cc Semiflex IC has a more rounded tip than the Farmer chamber, which is more of a thimble IC than a CIC. Future investigations with both the bigger 0.3 cc Semiflex IC and the Farmer chamber could explain the discrepancy between the experimental data and the analytical model.

Considering a possible clinical relevance, we extracted the physical dose of a biologically optimized carbon ion SOBP with a width of 6 cm and the energy of 350 MeV/u.^13^ The difference in physical dose over the 6‐cm SOBP was consistently 2%/mm, and the highest change in physical dose was 0.05 Gy over 1 mm at the distal dose gradient. Applied to the difference between the TRS‐398‐recommended *P*_eff_ and our *P*_eff_ of 0.3 mm, this means a change in dose of 0.7% over the entire SOBP. This can already exceed quality assurance thresholds for carbon ion treatment plans.

### 3.3 Validation of measurements with gamma analysis

Overall, the use of *P*_eff_ ≠ 0.75·*r*_cyl_ resulted in a lower mean gamma index and more points passing the acceptance criteria (see Table 4) than the use of *P*_eff_ = 0.75·*r*_cyl_. For carbon ions and the IAEA TRS‐398‐recommended *P*_eff_, six of eight failing points are in the dose fall‐off and can be explained by the steep dose gradient. One is in the beginning of the SOBP and one in the contamination tail. After the application of our best estimate using the information given in Table 3, *P*_eff_ = 0.86·*r*_cyl_, only four points fail the gamma analysis; all of which are located in the lower part of the dose fall‐off. For protons and the IAEA TRS‐398‐recommended *P*_eff_, again eight of 14 points fail the gamma analysis. They are located as follows: Two in the plateau, three in the SOBP, and three in the dose fall‐off. Using *P*_eff_ = 0.92·*r*_cyl_, the plateau points pass the gamma analysis while the other six points still fail. The remaining three failing points in the SOBP further demonstrate the previously mentioned fluctuations in the SOBP and the need for less strict passing criteria.

**TABLE 4:** Summary of the results of the gamma‐index analysis for the 0.125 cc Semiflex ionization chamber

	*P*_eff_	Mean gamma index	Failed points/Total points
Carbon ions	0.75·*r*_cyl_	1.00	8/14
	0.86·*r*_cyl_	0.84	4/14
Protons	0.75·*r*_cyl_	0.93	8/14
	0.92·*r*_cyl_	0.83	6/14

For carbon ions, the application of a factor to a dataset derived in part from the same dataset constitutes a limitation. However, *P*_eff_ was derived exclusively from the points in the dose fall‐off but was applied to all points including the plateau, SOBP, and contamination tail. Also, *P*_eff_ was derived from a second independent dataset and yielded a consistent result.

## 4 CONCLUSION

The accurate knowledge of *P*_eff_ is particularly important for measurements in proximity to high dose gradients such as in the distal fall‐off. Due to its high linear energy transfer, this region is of relevance for radiobiological or detector response experiments. Accurate *P*_eff_ is also of interest in precision radiation therapy.

In this study, *P*_eff_ of the 0.125 cc Semiflex IC was determined experimentally and differs from the IAEA TRS‐398 recommendation of 0.75·*r*_cyl_ (though agreement is within the standard uncertainty). The use of two independent methods provides robustness to the evaluation, and the improved gamma index through the application of the derived *P*_eff_ to the entire depth dose curve provides additional support for systematic difference. In proton dosimetry, no shift is recommended in absence of a dose gradient, which means that CICs are only recommended to be used in SOBPs. For depth dose measurements, only PPICs are recommended. We believe that CICs are also suitable for depth dose measurements, provided *P*_eff_ is known.

We recommend the use of the determined shifts at R80 to calculate the factor multiplied by the radius of the inner cavity of the 0.125 cc Semiflex IC (*r*_cyl_ = 2.75 mm), as it is the most utilized quality parameter for particle beams and gives a good estimate for the whole depth dose curve. For carbon ions, an average of both methods (gradient and peak‐to‐peak) yields (0.86 ± 0.10)·*r*_cyl_ while for protons, the peak‐to‐peak method yields (0.92 ± 0.08)·*r*_cyl_.

The increased agreement (lower gamma index) between measurements done in a proton beam and the TPS‐calculated dose, caused by using the independently derived *P*_eff_, demonstrates the need for more experimental determinations of *P*_eff_ for non‐Farmer‐type CICs in light ion beams.

## CONFLICT OF INTEREST

The authors have no conflict of interest to disclose.

## Data Availability

Research data are not shared.
